# Supplementary material for: Establishment and Application of Indirect ELISAs for Detecting Antibodies against Goose Astrovirus Genotype 1 and 2
Source: Vaccines (Basel). 2023 Mar 15;11(3):664. doi: 10.3390/vaccines11030664 (PMC10055997; doi:10.3390/vaccines11030664)
Supplement: Supplementary file 1 [file vaccines-11-00664-s001.zip › vaccines-2263633-supplementary.pdf]

Table S1. Determination of the coating virus concentration and serum dilution for the GAstV-1-ELISA.

Virus concentration (μg/mL)		Serum dilution 1:50	1:100	1:200	1:400	1:800
	P[a]	1.461	1.329	1.233	1.035	0.897
60	N[a]	0.324	0.207	0.179	0.209	0.126
	P/N	4.509	6.420	6.888	4.952	7.119
	P	1.16	1.168	1.056	1.062	0.902
30	N	0.166	0.183	0.120	0.123	0.097
	P/N	6.988	6.383	8.800	8.634	9.299
	P	1.019	1.227	1.144	1.037	0.874
20	N	0.185	0.115	0.117	0.102	0.086
	P/N	5.508	10.670	9.778	10.167	10.163
	P	0.965	1.226	1.039	1.092	0.945
15	N	0.156	0.152	0.136	0.106	0.089
	P/N	6.186	8.066	7.640	10.302	10.618
	P	1.278	1.221	1.212	1.101	0.934
12	N	0.204	0.137	0.127	0.099	0.086
	P/N	6.265	8.912	9.543	**11.121**	10.860
	P	1.035	1.014	0.998	0.997	0.793
10	N	0.229	0.126	0.106	0.101	0.076
	P/N	4.520	8.048	9.415	9.871	10.434

[a]The OD_{450} nm of positive (P) and negative (N) sera

Table S2. Determination of the coating protein concentration and sera dilution for the GAstV-2-Cap-ELISA.

Protein concentration (μg/mL)		Serum dilution 1:50	1:100	1:200	1:400	1:800	1:1600
	P[a]	1.42	1.34	1.16	1.08	0.90	0.71
10	N[a]	0.13	0.10	0.08	0.08	0.08	0.08
	P/N	11.32	13.84	14.13	13.71	11.12	8.55
	P	1.36	1.25	1.13	1.05	0.97	0.78
5	N	0.12	0.09	0.08	0.08	0.08	0.08
	P/N	11.62	13.70	14.13	13.40	12.40	9.62
	P	1.46	1.30	1.20	1.08	0.87	0.72
2.5	N	0.12	0.09	0.08	0.08	0.07	0.08
	P/N	12.36	14.26	15.64	13.72	11.80	9.17
	P	1.38	1.23	1.25	1.09	0.98	0.73
1.25	N	0.11	0.09	0.08	0.08	0.08	0.08
	P/N	12.63	14.27	**16.45**	14.51	12.59	9.43
	P	1.28	1.23	1.12	1.02	0.86	0.76
0.625	N	0.10	0.08	0.08	0.08	0.07	0.09
	P/N	12.32	15.16	14.57	13.53	11.78	8.27

[a]The OD_{450} nm of positive (P) and negative (N) sera.
